# Cross-sectional study of factors related to COVID-19 vaccination uptake among university healthcare students

**DOI:** 10.3389/fpubh.2023.1325942

**Published:** 2023-12-14

**Authors:** Akiko Kondo, Renaguli Abuliezi, Erika Ota, Tomomi Oki, Kazuko Naruse

**Affiliations:** ^1^International Nursing Development, Graduate School of Healthcare Sciences, Tokyo Medical and Dental University, Tokyo, Japan; ^2^Cognizant Japan, Tokyo, Japan; ^3^Global Health Nursing, St. Lukes International University, Tokyo, Japan; ^4^The Tokyo Foundation for Policy Research, Tokyo, Japan; ^5^School of Nursing and Rehabilitation Sciences, Showa University, Tokyo, Japan; ^6^School of Nursing, Tokyo Medical University, Tokyo, Japan

**Keywords:** vaccination uptake, COVID-19, healthcare students, preventive behavior, perceived control, resilience, Japan

## Abstract

**Introduction:**

Healthcare students are more likely to become infected than other university students as they may encounter patients with COVID-19 during clinical training. Vaccination uptake is essential to prevent infection. This study explored factors related to COVID-19 vaccination uptake among healthcare students.

**Methods:**

This cross-sectional study conducted online surveys of undergraduate and graduate nursing and healthcare graduate students from four medical universities in the Tokyo Metropolitan Area of Japan. Data were collected from June to August 2022, when the fourth vaccination program was initiated.

**Results:**

Data from 1,169 students were analyzed (response rate = 37.3%). The mean age was 25.1 ± 7.6 years, and most were female (82.3%). Academic majors included nursing (68.0%), medicine (16.3%), dentistry (9.3%), and others (6.4%). Thirty students (2.6%) were not vaccinated, one student (0.1%) had received one vaccination, 997 (85.3%) had received three, and 27 (2.3%) had received four. The major reason for not being vaccinated was insufficient confirmation of its safety (n = 25). Students who had received at least one vaccination (*n* = 1,139), 965 (84.7%) reported experiencing adverse side effects, the most frequent being pain at the injection site (76.2%), followed by fever (68.3%). In the logistic regression, a greater number of vaccinations (3–4 times) was associated with older age (odds ratio, OR = 1.53), working (OR = 1.67), and more frequent infection-preventive behaviors (OR = 1.05). Significantly fewer students were vaccinated at University B than at University A (OR = 0.46). Additionally, those majoring in subjects other than nursing (OR = 0.28), and students from non-Asian countries (OR = 0.30) were less likely to be vaccinated.

**Discussion:**

It is necessary to pay attention to and encourage the vaccination of students who engage in low levels of preventive behavior, students who are young, international, or unemployed, and those in non-healthcare professional majors.

## 1 Introduction

Nearly four years have passed since the coronavirus disease 2019 (COVID-19) was initially reported in Wuhan, China and the World Health Organization declared the spread of the disease to be a global pandemic. The number of cases in Japan ranked eighth in the world in terms of cumulative total cases by October 2023 (1). In Japan, vaccination was initially introduced for older persons in April 2021, which is relatively late compared to other countries, such as the United States and the United Kingdom, where vaccination efforts commenced in December 2020 (2). As of October 28, 2022, 80.43% of Japanese people were fully vaccinated, and the number of boosters per 100 people was 66.08, which is relatively high compared to other countries worldwide (3). However, COVID-19 variants continue to emerge (4); therefore, it is necessary to continue the vaccination program.

Studies have reported numerous factors that can affect vaccination intentions and behavior. Sociodemographic factors include gender (5); education, income, employment and political affiliation (6); race and ethnicity (7); and religiosity (8). Psychological factors, such as confidence in the vaccines’ importance (9), outbreak concerns and confidence in the government (5), the degree of government support (8), mistrust in information sources (10), societal and individual resilience (8), and experience of previous exposure to a patient with COVID-19 (11), also play a role. Other factors are self-rated health (11), seasonal flu vaccination (5), and knowledge of illness (5).

University students are young and active, and primarily it is thought to play a leading role in the transmission of SARS-CoV-2 (12, 13). Their major concerns regarding COVID-19 vaccination include its safety, efficacy, and limited information regarding the vaccine (14). Factors related to vaccination uptake among university students include their field of study (medical) and confidence in the safety and effectiveness of COVID-19 vaccines (15). Factors positively related to vaccination intention are knowledge, risk perception of COVID-19, perceived benefit of COVID-19 vaccination, and social norms (16). While female, non-white college students and healthcare workers were more likely to support mandatory influenza vaccination than other college students, COVID-19 vaccination mandate preferences were not statistically different among college students (14).

Healthcare students typically see patients with COVID-19 during clinical training and thus are more likely to become infected than other university students. Among medical residents, frontline residency, which entails direct involvement with COVID-19 patients, is a predictor of vaccination in the residency hospital (17). However, some students resist receiving the vaccine. According to an umbrella review (18), the vaccination acceptance rate among healthcare students ranged from 34–82%, and that among nursing and dental students approximated 60%.

The major determinants of vaccine hesitancy among healthcare students worldwide (18) were similar to those of other populations. These include sociodemographic factors (e.g., female sex, younger age, lower income, lower socioeconomic status, and being married); educational factors (e.g., dental students, non-medical students, or students in lower years); and health factors (e.g., perceived good health, unwillingness to accept influenza shots, lack of information about the vaccine, and insufficient knowledge about COVID-19). Other factors were a lower level of trust or confidence in the government, mass media, and social media; and social factors (e.g., absence of COVID-19 infection within the individual’s close social network). Additionally, vaccine hesitancy was associated with the following: previous COVID-19 infection, perceived low risk of severe COVID-19 now and in the future, concerns about vaccine safety, fear of adverse side effects, fee-based vaccines, insufficient knowledge about vaccination, and lower levels of trust or confidence in healthcare systems and agencies, health promotion strategies, public health experts, and scientists or pharmaceutical companies. In contrast, greater vaccine acceptance was associated with using scientists or pharmaceutical companies as COVID-19 vaccine information sources, suffering from distance from friends during pandemic isolation, and expressing a fear of death from the virus.

Vaccine acceptance levels differ among students residing in different countries (18). However, only one review out of 31 included healthcare students in Japan and reported the prevalence of COVID-19 vaccination among medical students (19). In Patwary et al.’s (20) country-specific analyses, the highest and lowest acceptance rates (88.0 and 66.2%) were in Romania and Iraq, respectively. However, no studies conducted in Japan were included in this review. One study reported that Japanese university students taking healthcare courses expressed significantly greater COVID-19 vaccination intention than those taking non-healthcare courses did (21). Among medical students at a university in Japan, factors related to willingness to receive a third dose of the COVID-19 vaccine were higher school year and confidence in the vaccine (22). However, the actual vaccination uptake status, reasons for not receiving the vaccine, factors associated with actual vaccine uptake among healthcare students (including dental and nursing students), and whether there exist differences among majors remain unknown.

Infection prevention behaviors are as important as vaccination prevents infection. Hyun et al. (23) found that receiving influenza vaccination predicted increased COVID-19 preventive behaviors among 141,902 adults in South Korea. In their review, Ripp et al. (24) reported belief in COVID-19-related conspiracy narratives was negatively associated with vaccination willingness and infection-preventive behavior. Similarly, we discovered that factors related to infection prevention behaviors among healthcare graduate students in the United States and Japan included nationality, sex, and perceived control (25). However, the association between perceived control, infection prevention behavior, and COVID-19 vaccination uptake remains unknown. Therefore, this study explored the factors related to COVID-19 vaccination uptake among healthcare students in Japan.

The following definitions are important in the context of this work. Perceived control is defined as “an individual’s subjective belief about the amount of control he or she has over the environment or outcome.” Conversely, “actual control describes the objective amount of control the individual has over the environment or outcome” (p. 254) (26). Studies have reported the importance of perceived control in infection prevention behaviors (25, 27, 28). In this study, perceived infection control was defined as the extent to which individuals felt that the COVID-19 infection was controlled. Resilience is a psychological concept defined as an individual’s ability to utilize strategies to cope with and grow as a result of stress or adversity (29, 30). Finally, infection prevention behaviors are health behaviors that aim to prevent COVID-19 infection (25).

## 2 Methods

### 2.1 Study design and participants

This was a cross-sectional study that used online surveys. The participants were undergraduate and graduate nursing and healthcare graduate students at four medical universities in the Tokyo Metropolitan area, Japan who voluntarily agreed to participate.

### 2.2 Data collection

We employed Google Forms (in both Japanese and English) for the survey so that both Japanese and international students could participate. Participants were provided with verbal and/or written information about the study at the end of classes and/or received e-mail invitations to access a form explaining the purpose and methods of the anonymous survey. Participants indicated that they had read the information and consented to participate in the study by clicking the “Agree” button on the survey form. Data were collected from June to August 2022, when the fourth vaccination program began in Japan. Healthcare students could receive free vaccinations at affiliated university hospitals.

### 2.3 Survey contents

The questionnaire queried the number of COVID-19 vaccinations received, reasons for not receiving the vaccine, vaccination side effects, sociodemographic data (11 items), experience of having COVID-19 (four items), psychological effects of the pandemic (four items), knowledge of COVID-19 and vaccination (two items), perceived infection control (three items), resilience (21 items), and individual infection-preventive behaviors (16 items) (See Supplementary File). These items are related to vaccination uptake reported in previous studies. To validate the contents of the questions, all authors, who were nursing professionals, discussed the contents. To test face validity, eight nursing graduate students were asked for pilot test and revised unclear questions several times.

The number of vaccinations received ranged from zero to four. Sociodemographic data included age, sex, chronic conditions requiring regular checkups, academic status (undergraduate or graduate), housing situation (i.e., roommate/housemate or none), religious affiliations, work status (i.e., full-time, part-time, or unemployed), university, major, and nationality. Experience with COVID-19 included COVID-19 infection (self or family) and having experience with COVID-19 patients.

The psychological effects of the pandemic were probed by asking “To what degree has the COVID-19 pandemic affected your daily life and work (including part-time job/study)?,” “To what degree has the COVID-19 pandemic affected you financially?,” “To what degree are you worried about academic (study/research) delays due to the COVID-19 pandemic?” and “To what degree do you have the confidence to overcome the impact of COVID-19 on your life?” Each question was answered using a four-point Likert scale (4 = strongly, 3 = moderately, 2 = slightly, and 1 = not at all). Knowledge of COVID-19 and vaccination were rated using a five-point Likert scale (5 = extremely high, 4 = high, 3 = moderate, 2 = low, or 1 = extremely low).

Perceived infection control was measured at three levels: individual, university, and the area (community) in which participants lived. For each level, the following questions were posed: “How much do you control to avoid viral infection? (1 = no control, 2 = a little control, and 3 = a great deal of control),” “Do you think your university has adequate COVID-19 infection prevention management? (5 = strongly agree, 4 = agree, 3 = uncertain, 2 = disagree, and 1 = strongly disagree),” and “Do you think that COVID-19 infection prevention management is adequate in your area? (5 = strongly agree, 4 = agree, 3 = uncertain, 2 = disagree, and 1 = strongly disagree).”

Resilience was measured using Hirano’s Bidimensional Resilience Scale (31), which was developed based on Cloninger’s temperament/character model to separately consider the innate and acquired factors of resilience (32). Resilience consists of seven factors: four innate resilience factors (optimism, control, sociability, and vitality) and three acquired resilience factors (attempting to solve a problem, self-understanding, and understanding others). Each factor is assessed via three questions, with 21 questions in total. Each question is answered on a five-point scale (5 = strongly agree, 4 = agree, 3 = neither disagree nor agree, 2 = disagree, and 1 = strongly disagree). One item is reverse-scored, and scores are summed to provide a total score that ranges from 21 to 105, with higher scores indicating greater resilience. The bidimensional structure and validity of the scale have been examined among Japanese people using higher-order factor analysis and a comparison of relevance with existing measures. Cronbach’s alpha for the scale was previously reported to be 0.90 (31). The scale is freely available without permission from creator’s home page (33).

Finally, the individual infection-preventive behavior questionnaire comprised 16 items related to preventive actions to decrease the transmission of COVID-19 (e.g., wearing a mask in public and avoiding crowded, closed, and close-contact settings). Participants were asked to respond to each item by selecting one option from a four-point categorical scale (3 = always, 2 = often, 1 = sometimes, and 0 = never). The total score for the preventive health behavior questions ranged from 0 to 48, with higher scores indicating more frequent practice of preventive behaviors. The first and second authors created health behavior questionnaires based on the Japanese government’s recommendations for preventive protocols of the Ministry of Health Labor and Welfare and Kamenidou et al.’ s (34) questions (25). One question—“Clean and disinfect shared objects and surfaces”—was excluded in this study because we considered it unnecessary. We also consulted an external expert. The other authors, who are nursing professionals in Japan, confirmed the content and agreed that the questions covered the necessary preventive health behaviors. Cronbach’s alpha of the preventive health behaviors questionnaire was good (0.843) in this study.

### 2.4 Sample size and power

G-Power 3.1.9.7 (35) was used to calculate the required sample size. For a Spearman correlation analysis with an effect size of 0.10 or 0.15, 1,289 or 565 participants were required, respectively, given an alpha level of 0.05 (two tailed) and power of 0.95. A total of 3,131 students were invited to participate in this study.

### 2.5 Analysis

SPSS version 28 (IBM, 2022) was used for the analysis. Data duplication was detected using SPSS. The factors related to the number of vaccinations received at an alpha level of 0.05 in bivariate analyses were entered into the logistic regression (0–2 or 3–4 vaccinations). Spearman’s correlation coefficients were used to determine the association between the number of vaccinations and ordinal or continuous variables (e.g., age, perceived control, and preventive behaviors). The Mann–Whitney U test or Kruskal-Wallis test with Bonferroni adjustment was performed to compare ordinal variables (e.g., number of vaccinations received and perceived knowledge) between two groups (e.g., graduate or undergraduate program) and three or more groups (e.g., four age groups, university, nationality, major), respectively. The association between the side effects of vaccination and number of vaccinations was analyzed among those who had received at least one vaccination. A chi-square test was used to determine the association between nominal variables (e.g., work, academic programs, international students, and religiosity). Logistic regression model fit was assessed using the Hosmer and Lemeshow test (*p* > 0.05) and variance inflation factors (< 2.0).

### 2.6 Ethical considerations

This study was approved by the research ethics committees of all participating universities (M2021-231). We explained the voluntary nature of the study, data confidentiality, and that answers would not affect respondents’ grades. To thank the participants for their time, those who submitted their e-mail addresses were sent Amazon gift cards worth 500 yen (approximately 3.3 USD). E-mail addresses were collected through a separate Google Form that was not connected to the survey form.

## 3 Results

### 3.1 Participant characteristics

A total of 1,176 datasets were submitted, of which seven were duplicated and excluded. Data of 1,169 students were used for the analyses (response rate = 37.3%; Table 1). The mean age was 25.1 ± 7.6 years, and most (82.3%) respondents were female. Academic majors included nursing (68.0%), medicine (16.3%), dentistry (9.3%), and others (6.4%), including medical administration, public health, biomedical sciences and engineering, and health policy science. More than half of participants were undergraduate students (55.9%). One hundred and sixteen (9.9%) participants were international students. Nationalities included Japan (n = 1,043; 89.2%), China (n = 45; 3.8%), Hong Kong (n = 1; 0.1%), other Asian countries (n = 49; 4.2%), and non-Asian countries (n = 18; 1.5%), including Africa (n = 16) and the United States (n = 2). Most (72.8%) worked (full- or part-time).

**Table 1:** Participants’ sociodemographic factors, experience with COVID-19, and association with number of vaccinations received (*N* = 1,169).

Items	Category	*n* (%)	Number of vaccinations received		*p* value
			Mean	Median	
Age (years)	≤19	259 (22.2)	2.74	3	<0.001^a^
	20–29	601 (51.4)	2.89	3	
	30–39	199 (17.0)	2.87	3	
	≥40	67 (5.7)	2.85	3			Missing	43 (3.7)			
Sex	Male	197 (16.9)	2.80	3	0.313^a^
	Female	962 (82.3)	2.86	3	
	Others	10 (0.9)	2.80	3	
Chronic conditions requiring regular check-ups	Yes	126 (10.8)	2.90	3	0.104^b^
	No	1,043 (89.2)	2.84	3	
Academic programs	Undergraduate	653 (55.9)	2.86	3	0.640^b^
	Graduate	516 (44.1)	2.83	3	
Roommate/housemate	Yes	844 (72.2)	2.85	3	0.649^b^
	No	312 (26.7)	2.86	3	
	Missing	13 (1.1)			
Religious affiliation	Yes	281 (24.0)	2.90	3	0.180^b^
	No	856 (73.2)	2.84	3	
	Missing	32 (2.7)			
Working	Yes	851 (72.8)	2.88	3	<0.001^b^
	No	300 (25.7)	2.76	3	
	Missing	18 (1.5)			
University	A	581 (49.7)	2.83	3	<0.001^a^
	B	208 (17.8)	2.78	3	
	C	112 (9.6)	2.95	3	
	D	268 (22.9)	2.88	3	
Major	Nursing	795 (68.0)	2.86	3	<0.001^a^
	Medical	190 (16.3)	2.90	3	
	Dental	109 (9.3)	2.83	3	
	Others	75 (6.4)	2.61	3	
Nationality	Japan	1,043 (89.2)	2.86	3	0.009^a^
	China or Hong Kong	46 (3.9)	2.76	3	
	Other Asia	49 (4.2)	2.96	3	
	Non-Asia	18 (1.5)	2.72	3	
	Missing	13 (1.1)			
International students	Yes	116 (9.9)	2.79	3	0.041^b^
	No	1,053 (90.1)	2.85	3	
COVID-19 infection (self)	Yes	116 (9.9)	2.84	3	0.864^b^
	No	1,041 (89.1)	2.85	3			Missing	12 (1.0)			
COVID-19 infection (family)	Yes	630 (53.9)	2.86	3	0.842^b^
	No	523 (44.7)	2.85	3			Missing	16 (1.4)			
Having experience with COVID-19 patients	Yes	202 (17.3)	2.84	3	0.549^b^
	No	954 (81.6)	2.86	3			Missing	13 (1.1)			
Continuous experience with COVID-19 patients	Yes	31 (2.7)	2.81	3	0.781^b^
	No	1,120 (95.8)	2.86	3			Missing	18 (1.5)			

Number of vaccinations received ranges from 0 to 4.

a Kruskal-Wallis test.

b Mann–Whitney U test.

Thirty students (2.6%) had not been vaccinated, one student (0.1%) had received one vaccination, 114 (10.0%) had received two vaccinations, 997 (85.3%) had received three, and 27 (2.3%) had received four vaccinations. The main reason for not receiving vaccination was insufficient confirmation of its safety (n = 25), followed by concerned about vaccine side effects (n = 15; Table 2). Among students who had been vaccinated at least once (n = 1,139), 965 (84.7%) reported experiencing side effects. The most frequent side effect was pain at the injection site (76.2%), followed by fever (68.3%; Table 3).

**Table 2:** Reasons not to receive vaccination (*N* = 30; multiple choice).

Reason	*n* (%)
Insufficient safety confirmation	23 (76.7)
Concerned about side effects of the vaccine	15 (50.0)
I’m in a low-risk group.	6 (20.0)
I will not get seriously ill from COVID-19.	3 (10.0)
I am allergic to vaccines.	2 (6.7)
I do not like injections/needles.	1 (3.3)
I’ve had a COVID-19 infection, so I am likely have antibodies to the disease.	1 (3.3)

**Table 3:** Side effects of vaccination and association with number of vaccinations received (*N* = 1,139; multiple choice).

Side effect	Yes/No	*n* (%)	Number of vaccinations received		*p* value
			mean	median	
Experienced at least one side effect	Yes	965 (84.7)	2.92	3	0.609
	No	174 (15.3)	2.91	3	
Pain on the arm	Yes	868 (76.2)	2.92	3	0.775
	No	271 (23.8)	2.92	3	
Fever	Yes	778 (68.3)	2.93	3	0.412
	No	361 (31.7)	2.91	3	
Swelling on the arm	Yes	599 (52.6)	2.92	3	0.886
	No	540 (47.4)	2.92	3	
Tiredness	Yes	531 (46.6)	2.93	3	0.249
	No	608 (53.4)	2.91	3	
Headache	Yes	504 (44.2)	2.93	3	0.293
	No	635 (55.8)	2.91	3	
Nausea	Yes	40 (3.5)	2.90	3	0.693
	No	1,099 (96.5)	2.92	3	
Diarrhea	Yes	23 (2.0)	2.91	3	0.912
	No	1,116 (98.0)	2.92	3	

Number of vaccinations received ranges from 0 to 4.

Mann–Whitney U test.

Knowledge of both COVID-19 (*p* < 0.001) and vaccination (*p* = 0.005) were better among graduate students (3.30 ± 0.69 and 3.23 ± 0.71, respectively) than among undergraduate students (3.17 ± 0.57 and 3.12 ± 0.59, respectively). Medical students (3.36 ± 0.67 and 3.31 ± 0.71, respectively) had significantly greater knowledge than nursing (3.19 ± 0.60 and 3.13 ± 0.61, respectively) and dental students (3.17 ± 0.73 and 3.15 ± 0.69, respectively) of both COVID-19 (*p* = 0.001 and *p* = 0.013, respectively) and vaccination (*p* = 0.002 and *p* = 0.040, respectively).

Regarding associations with demographic factors, University C had significantly lower scores for perceived control (“Do you think your university has adequate COVID-19 infection prevention management?”) compared with the other universities (*p* < 0.05). There was no difference in the ratio of students who were working between graduate (72.3%) and undergraduate (75.2%) students (*p* = 0.257). International students (52.2%) were significantly more religious than Japanese students (21.7%; *p* < 0.001).

### 3.2 Factors related to receiving vaccination

The number of vaccinations differed significantly among age groups (*p* < 0.001), universities (*p* < 0.001), and majors (*p*  < 0.001; Table 1). Students under 20 years of age had received significantly fewer vaccinations compared to those aged 20–29 years (*p*  < 0.001), 30–39 years (*p*  < 0.001), and 40 years and older (*p* = 0.025). Students in the other majors group had received significantly fewer vaccinations compared to those taking different, discrete majors (*p* < 0.001). Japanese students had received significantly more vaccinations than non-Asian students (*p* = 0.040) and students from China and Hong Kong (*p* = 0.029). Other Asian students had received more vaccinations than non-Asian students (*p* = 0.010) or students from China and Hong Kong (*p* = 0.008). Overall, international students had received significantly fewer vaccinations than Japanese students (*p* = 0.041). Employed students had received significantly more vaccinations than unemployed students (*p* < 0.001).

Table 4 presents the Spearman’s correlation coefficients. Students who strongly thought that their university had adequate COVID-19 infection prevention management (ρ = 0.067, *p* = 0.021) and students with higher preventive behaviors scores received more vaccinations (ρ = 0.070, *p* = 0.016). While preventive behaviors were positively related to individual (ρ = 0.379, *p* < 0.001), university (ρ = 0.157, *p* < 0.001), and community (ρ = 0.083, *p* = 0.004) levels of perceived control, there was no significant association between perceived control and the number of vaccinations received. While preventive behaviors were also positively related to resilience total score (ρ = 0.146, *p* < 0.001) and innate (ρ = 0.098 *p* = 0.001) and acquired (ρ = 0.187, *p* < 0.001) resilience subscale scores, there was no significant association between resilience and the number of vaccinations received.

**Table 4:** Correlation with number of vaccinations received.

	1	2	3	4	5	6	7	8	9	10	11	12	13
1. Number of vaccinations received	1												
2. COVID-19 pandemic affected daily life/work/study	0.250	1											
3. COVID-19 pandemic affected finances	−0.010	0.282^***^	1										
4. Worried about academic delays	0.031	0.249^***^	0.397^***^	1									
5. Confidence overcoming the impact of COVID-19 on life	−0.045	0.001	0.071^*^	−0.072^*^	1								
6. Knowledge of COVID-19	0.016	0.050	0.023	0.045	0.122^***^	1							
7. Knowledge of COVID-19 vaccine	0.014	0.015	−0.009	0.022	0.070^*^	0.740^***^	1						
8. Avoid COVID-19 infection	0.033	0.111^***^	0.029	0.070^*^	0.067^*^	0.102^***^	0.112^***^	1					
9. University’s COVID-19 infection prevention management	0.067^*^	0.066^*^	−0.021	−0.011	0.126^***^	0.088^**^	0.084^**^	0.129^***^	1				
10. Area’s COVID-19 infection prevention management	−0.010	0.000	0.022	−0.016	0.200^***^	0.080^**^	0.105^***^	0.057	0.403^***^	1			
11. Resilience total score	−0.042	0.055	0.008	−0.013	0.215^***^	0.260^***^	0.228^***^	0.153^***^	0.160^***^	0.128^***^	1		
12. Innate resilience subtotal	−0.041	0.030	0.013	−0.024	0.207^***^	0.214^***^	0.189^***^	0.097^**^	0.146^***^	0.125^***^	0.932^***^	1	
13. Acquired resilience subtotal	−0.024	0.069^*^	−0.008	0.006	0.180^***^	0.261^***^	0.237^***^	0.212^***^	0.156^***^	0.102^***^	0.831^***^	0.589^***^	1
14. Preventive behavior total score	0.070^*^	0.190^***^	0.142^***^	0.170^***^	−0.007	0.061^*^	0.045	0.379^***^	0.157^***^	0.083^**^	0.146^***^	0.098^***^	0.187^***^

Spearman’s correlation coefficients: *p < 0.05, **p < 0.01, ***p < 0.001.

There were no significant associations between the number of vaccinations and other sociodemographic factors (sex, chronic conditions, academic status, roommate/housemate, religious affiliations), experience with COVID-19 (Table 1), psychological effects of the pandemic, perceived infection control at the community or individual level, perceived knowledge of COVID-19 or vaccination (Table 4), side effects of vaccination, or each type of side effect (Table 3).

In the logistic regression (Table 5) receiving more vaccinations (3–4 times) was associated with older age (odds ratio, OR = 1.53, 95% confidence interval, CI = 1.11–2.12), working (OR = 1.67, 95% CI = 1.10–2.54), and more frequent infection-preventive behaviors (OR = 1.05, 95% CI = 1.02–1.08). Fewer vaccinations were associated with students in University B compared with University A (OR = 0.46, 95% CI = 0.24–0.87), other majors compared a nursing major (OR = 0.28, 95% CI = 0.13–0.59), and students from non-Asian countries compared with Japanese students (OR = 0.30, 95% CI = 0.09–0.99).

**Table 5:** Factors related to vaccination uptake according to logistic regression.

	B	Exp(B)	(95%CI)	*p*	VIF
Age (10-year intervals)	0.428	1.533	(1.109–2.120)	0.010	1.395
Nationality: Japanese (reference)					
China or Hong Kong	−0.614	0.541	(0.224–1.306)	0.172	1.149
Other Asia	−0.438	0.645	(0.257–1.620)	0.351	1.272
Non-Asia	−1.210	0.298	(0.090–0.988)	0.048	1.054
University A (reference)					
B	−0.780	0.458	(0.243–0.865)	0.016	1.770
C	1.021	2.776	(0.897–8.585)	0.076	1.467
D	−0.212	0.809	(0.413–1.586)	0.538	1.827
Their university has adequate COVID-19 infection prevention management	0.158	1.171	(0.930–1.476)	0.180	1.087
Major: Nursing (reference)					
Medical	0.027	1.027	(0.466–2.262)	0.947	1.836
Dental	−0.263	0.768	(0.328–1.800)	0.544	1.715
Other	−1.285	0.277	(0.129–0.591)	0.001	1.323
Employed (vs. unemployed)	0.512	1.669	(1.096–2.540)	0.017	1.129
Preventive behaviors	0.049	1.050	(1.021–1.080)	0.001	1.109

Outcome: vaccination uptake 3–4 times = 1, 0–2 times = 0.

Nagelkerke R2 = 0.133; Hosmer and Lemeshow test *p* = 0.599.

VIF, variance inflation factor.

## 4 Discussion

Most participants had received three or more vaccinations. The major reasons for not receiving the vaccine were insufficient safety confirmation and concerns regarding its side effects. The major side effects of vaccination were pain, fever, and swelling of the arm. Factors related to COVID-19 vaccination uptake among healthcare students were sociodemographic factors (e.g., age, work status, major, university, nationality) and individual infection-preventing behaviors. The psychological effects of the pandemic, knowledge of COVID-19 and vaccination, perceived infection control, resilience, and side effects were not related to vaccine uptake.

This is the first study to report an association between infection-preventive behaviors and vaccination uptake. Students with a higher level of infection-preventive behaviors had greater vaccination uptake. Faasse et al. (5) found that higher knowledge about COVID-19 predicted decreased engagement with health-protective behaviors but higher vaccination intentions among the general Australian population. It is possible that students who received vaccinations may not have engaged in more preventive behaviors because they thought vaccination would prevent infection. However, this was not the case for university healthcare students in this study. Healthcare students who considered infection prevention important, those who perceived COVID-19 as a risk, and those who perceived the benefits of COVID-19 vaccination (16) may have opted to be vaccinated.

Increased vaccination uptake was associated with older age; nursing, medical, and dental education compared to other majors; and working. Older students working as health professionals may have been provided with early vaccination opportunities or have been subject to mandatory vaccination by their employers (17). More students majoring in medical professionals received vaccinations, which agrees with the previous studies (15) (36).

After adjusting for other factors, we determined that international students from all areas, especially non-Asian countries, received fewer vaccinations than Japanese students did (OR < 1). Ethnic disparity is obvious in the United States and Nguyen et al. considered the role of minority populations’ limited accessibility to vaccines (7). Although information emails were provided in both English and Japanese in University A, international students who do not understand Japanese may have had limited access to vaccination opportunities, In Japan, social and healthcare systems for non-Japanese people are insufficient, and international students in Japan have difficulties accessing healthcare due to language barriers. Some have expressed a desire for more English-speaking Japanese healthcare staff (37).

Students at University B had significantly lower vaccination uptake, but responses to the university level perceived control question did not differ from those of students at the other universities. In addition, this question was not significantly related to vaccination uptake after adjusting for other variables. In University B, fourth vaccinations were provided off campus. Offering free on-site vaccination was the most successful tool to ensure vaccination adherence (38). One reason for the lower vaccination rate at University B may be the difference in accessibility to students. In this study, a higher level of perceived control was related to a higher level of preventive behaviors, which agreed with the results of our previous study (25), and a higher level of preventive behaviors was related to increased vaccination uptake. However, perceived control at any level (individual, university, or community) was not directly related to vaccination.

Among the demographic factors, sex, chronic conditions, academic status, roommate/housemate status, and religious affiliation were not related to vaccination uptake. While male sex was associated with lower engagement with health-protective behaviors, female sex significantly predicted a lower likelihood of being vaccinated against COVID-19 (5). In our study, female students reported receiving a slightly higher number of vaccinations, but the difference was not statistically significant. However, most students were female, which could have decreased the power to detect differences between the sexes. Young adults (11) and healthcare students (18) who rated themselves as having better health had lower vaccination intentions or acceptance. In our study, participants with chronic conditions requiring regular checkups tended to have more vaccinations, but the number was not high (10.8%), and the study power may have been insufficient to detect the difference. There was no difference in the vaccination uptake between the undergraduate and graduate students in our study. Although undergraduate students are not yet professional healthcare workers and their perceived knowledge about COVID-19 and vaccination is lower than that of graduate students, working individuals may realize the importance of vaccination to avoid infection (9). Kimhi et al. (8) reported that a higher level of religiosity was associated with lower vaccine uptake but not with vaccine hesitancy. In Japan, the proportion of religious people is low, especially among young people, and the effect of religiosity may not be marked (25).

Experience with COVID-19, the psychological effects of the pandemic, knowledge of COVID-19 and vaccination, resilience, and the side effects of vaccination were not related to the number of vaccinations. Even though students were not forced to take the vaccination, social norms (16) may have influenced their decision on whether to get vaccinated. Therefore, previously reported factors may not be related to vaccination uptake. In addition, healthcare students in this study were able to receive vaccination at their affiliated hospital, where treatments for side effects had been prepared. Therefore, even though they were concerned about side effects, they may have decided to receive. According to an umbrella review (18), both actual and perceived knowledge about COVID-19 were positively related to vaccine acceptance among healthcare students. We measured perceive knowledge of COVID-19 and vaccination, which may have been relatively similar among the healthcare students. Additionally, although the major reason for not getting vaccinated was insufficient safety confirmation, very few students (n = 23) reported this concern. Furthermore, although the second most frequent reason for not receiving vaccination was concerns about the vaccine’s side effects, none of side effects experienced was significantly related to the number of vaccinations among students who received the vaccine at least once. Kimhi et al. reported that individual resilience was negatively associated with vaccine hesitancy but not vaccine uptake (8). These non-significant factors may also be related to vaccination intention rather than actual vaccination uptake.

The World Health Organization developed the Behavioral and Social Drivers (BeSD) vaccination model. According to this model, confidence affects motivation, which, in turn, impacts vaccination acceptance behavior (39, 40). Confidence includes the aspects of thinking and feeling (perceived disease risk and vaccine confidence) and social processes (social norms, and health worker recommendations). Practical issues (availability, affordability and ease of access) also moderate the process from motivation to vaccination uptake (40, 41). In our study, low vaccine confidence and low perceived disease risk were major reasons to not get vaccinated. Social process, such as nationality, belonging university, majors and working status, were the main predictors of vaccination uptake. Although we did not collect motivation variables, our study results suggest that more attention needs to be paid to international students’ accessibility to vaccines.

### 4.1 Limitations and further studies

The cross-sectional design restricted our ability to establish causality, and self-reported data may have introduced bias. This study collected data from four universities in the Tokyo Metropolitan area, Japan, approximately half of which were for undergraduate nursing students. Consequently, our results cannot be generalized to all healthcare students in Japan. The number of international students in universities is low, especially in non-Asian countries. Therefore, these results should be interpreted with caution. It is necessary to conduct a larger survey with more universities, healthcare majors, and international students. Since vaccination uptake can be influenced by organizational plans and social norms, it is necessary to explore the factors related to healthcare students’ vaccination intentions. Future research should focus on 1) understanding the specific factors influencing vaccination decisions, including motivation and accessibility for international healthcare students and 2) developing targeted interventions to address vaccine hesitancy. Longitudinal studies can provide insights into changes in vaccination behavior over time and the impact of public health campaigns on healthcare student populations. It is also necessary to follow the status of fifth and more vaccination uptake for vaccines not offered on campus and those requiring payment in terms of availability and affordability (41).

### 4.2 Practical implications

The findings of this study have important implications for public health efforts targeting healthcare students. It is necessary to pay attention to and encourage the vaccination of students with low levels of preventive behaviors; young, international, and unemployed students; and students in non-healthcare professional majors. It is necessary to provide information in English, their native language or non-complicated, easily understandable languages regarding vaccination to international students so that they can understand the necessity of vaccination and ascertain where and how they can receive vaccination. Providing clear and accessible information about vaccine safety and efficacy and addressing university- or major-specific disparities are critical steps toward achieving higher vaccination rates within this population.

## 5 Conclusion

Demographic factors, such as nationality, university, academic major, and age, were the main factors related to vaccination uptake. Students who were employed and engaged in infection-preventive behaviors received more vaccinations. The side effects of vaccination, knowledge, resilience, and perceived infection control were not related to vaccination uptake. It is necessary to pay attention to and encourage the vaccination of students with low levels of preventive behaviors; young, international, and non-working students; and students in non-healthcare professional majors.

## Data availability statement

The raw data supporting the conclusions of this article will be made available by the authors, without undue reservation.

## Ethics statement

The studies involving humans were approved by Medical Research Ethics Committee at the Tokyo Medical and Dental University, which was commissioned by Tokyo Medical University (Approval number: M2021-231). St. Luke’s International University Research Ethics Review Committee (Approval number: 22-A024). Ethics committee of Showa University (Approval number: 22-034-B). The studies were conducted in accordance with the local legislation and institutional requirements. The ethics committee/institutional review board waived the requirement of written informed consent for participation from the participants or the participants' legal guardians/next of kin because it was an anonymous survey and risk was minimal.

## Author contributions

AK: Conceptualization, Data curation, Formal analysis, Funding acquisition, Writing – original draft, Writing – review & editing. RA: Conceptualization, Data curation, Investigation, Writing – review & editing. EO: Data curation, Investigation, Writing – review & editing. TO: Data curation, Investigation, Writing – review & editing. KN: Data curation, Investigation, Writing – review & editing.
