# Supplementary material for: Cross-sectional study of factors related to COVID-19 vaccination uptake among university healthcare students
Source: Front Public Health. 2023 Dec 14;11:1325942. doi: 10.3389/fpubh.2023.1325942 (PMC10752956; doi:10.3389/fpubh.2023.1325942)
Supplement: Supplementary file 1 [file Data_Sheet_1.PDF]

「新型コロナウイルス流行下での医療系大学生の健康行動とコントロール感、レジリエンスとの関係について国際比較」“The relationship between Health Behaviors, Perceived Control, and Resilience of Health Professional Students under covid-19 pandemic: an international comparison”

* Required

ワクチン関する質問項目 Questions regarding the COVID-19 vaccine COVID-19

1. コロナウイルスのワクチンを接種しましたか？ Have you been vaccinated for the coronavirus? *

   Mark only one oval.

   - はい,4回 Yes, 4 times
   - はい,3回 Yes, 3 times
   - はい2回 Yes, 2 times
   - はい1回 Yes, 1 time
   - いいえ No

ワクチン接種したくない理由について Reason for not being vaccinated

2. 接種していないと回答した方について、接種しない理由は何ですか？（複数回答可）What is the reason for not being vaccinated? (Multiple answers) *

   Check all that apply.

   - [ ] 安全性の確認が不十分 Insufficient safety confirmation
   - [ ] 副作用が心配 I would be concerned about side effects from the vaccine
   - [ ] 注射/針が嫌い I don’t like injection/needles
   - [ ] 自分はリスクが低いグループなので、接種しなくても良い I’m in a low-risk group for getting seriously ill from COVID-19
   - [ ] 感染しても重症化しないと思う I’m in a low-risk group for getting seriously ill from COVID-19
   - [ ] 過去に感染したので、抗原を持っていると思う I’ve had a COVID-19 infection, so I likely have antibodies to the dis-ease
   - [ ] 接種してもワクチンは有効ではないと思う I don’t think vaccines work well
   - [ ] ワクチンにアレルギーがある I am allergic to vaccines
   - [ ] 接種する機会がなかった I didn't have a chance to inoculate

   Other: [ ] ______________________

副作用について About side effects

3. 接種したと回答した方について、何か副作用がありましたか？ Did you have any side effects after being vaccinated? *

Mark only one oval.

- はい Yes
- いいえ No

Covid-19ワクチンの副作用 Side effect of covid-19 vaccine

4. 副作用があった方について、どのような副作用がありましたか？（複数回答可）For those who had side effects, what were the side effects? (Multiple answers) *

Check all that apply.

- [ ] 接種部の痛み Pain on the arm where you got the shot
- [ ] 接種部位の腫れ Swelling on the arm where you got the shot
- [ ] 疲労感 Tiredness
- [ ] 頭痛 Headache
- [ ] 発熱 Fever
- [ ] 下痢 Diarrhea
- [ ] 吐き気・嘔吐 Nausea

Other: [ ] ______________________

追加でコロナウイルスのワクチンの接種について Additional coronavirus vaccination

5. さらにコロナウイルスのワクチンの接種が必要になったら受けますか？ If you need to get an additional coronavirus vaccine, will you get it? *

Mark only one oval.

- 是非受けたい Definitely want to receive it
- 必要なら受ける Receive if necessary
- あまり受けたくない Not really want to receive
- 受けたくない Don't want to receive

追加で接種に関する質問 Additional coronavirus vaccine

6. 「受けたくない」と「あまり受けたくない」 を選んだ方について理由を教えて下さい。（複数回答）Why did you choose this answer? (if answered “Don't want to receive” or “Not really want to receive” for the above question) (Multiple answers) *

Check all that apply.

- [ ] 前回接種した副作用が発症した Had side effects of the previous inoculation
- [ ] 安全性の確認が不十分 Insufficient safety confirmation
- [ ] 注射/針が嫌い I don’t like injection/needles
- [ ] 追加で接種しなくても予防できる Can be prevented without additional inoculation
- [ ] 既に摂取しているので感染しても重症化しないと思う I’ve had a COVID-19 infection, so I likely have antibodies to the dis-ease
- [ ] 過去に感染したので、抗原を持っていると思う I’ve had a COVID-19 infection, so I likely have antibodies to the disease
- [ ] 接種してもワクチンは有効ではないと思う I don’t think vaccines work well
- [ ] ワクチンにアレルギーがある I am allergic to vaccines

Other: [ ] ______________________

Covid-19に関する知識について Knowledge of covid-19

7. コロナウイルス（SARS-CoV-2）とそれが引き起こすCOVID-19という病気についての私の知識は次のとおりです。My knowledge of severe acute respiratory syndrome coronavirus 2 (SARS-CoV-2) and the COVID-19 disease is: *

Mark only one oval.

- ( ) 非常に低い Extremely low
- ( ) やや低い Low
- ( ) 適度 Moderate
- ( ) やや高い High
- ( ) 非常に高い Extremely high

8. COVID-19のワクチンに関する私の知識は次のとおりです。 My knowledge of COVID-19 vaccine is: *

Mark only one oval.

- 非常に低い Extremely low
- やや低い Low
- 適度 Moderate
- やや高い High
- 非常に高い Extremely high

二次元レジリエンス要因尺度 Bidimensional Resilience Scale (BRS)

あなた自身についてお答えください。以下の21項目について、1「まったくあてはまらない」～5「よくあてはまる」の中で、もっとも当てはまると思う回答を一つ選んでください。
The following 21 statements concern your perception about yourself in a variety of situations. Please indicate the strength of your agreement with each statement, utilizing a scale in which 1 denotes strong disagreement, 5 denotes strong agreement, and 2, 3, and 4 represent intermediate judgments. Please choose one strength of consent for each statement.

9. 1. どんなことでも、たいてい何とかなりそうな気がする。 I think that things will work out on most occasions in any case. *

Mark only one oval.

- よくあてはまる Strongly agree
- ややあてはまる Agree
- どちらともいえない Neither disagree nor agree
- あまりあてはまらない Disagree
- まったくあてはまらない Strongly disagree

10. 2. 昔から、人との関係をとるのが上手だ。 I am good at preserving friendships since I was a child. *

Mark only one oval.

- よくあてはまる Strongly agree
- ややあてはまる Agree
- どちらともいえない Neither disagree nor agree
- あまりあてはまらない Disagree
- まったくあてはまらない Strongly disagree

11. 3. 自分の性格についてよく理解している。I understand my personality well. *

Mark only one oval.

- よくあてはまる Strongly agree
- ややあてはまる Agree
- どちらともいえない Neither disagree nor agree
- あまりあてはまらない Disagree
- まったくあてはまらない Strongly disagree

12. 4. たとえ自信がないことでも、結果的に何とかなると思う。 I think that things will work out finally even if I have no confidence. *

Mark only one oval.

- よくあてはまる Strongly agree
- ややあてはまる Agree
- どちらともいえない Neither disagree nor agree
- あまりあてはまらない Disagree
- まったくあてはまらない Strongly disagree

13. 5. 自分から人と親しくなることが得意だ。 It is good to be intimate with another person. *

Mark only one oval.

- よくあてはまる Strongly agree
- ややあてはまる Agree
- どちらともいえない Neither disagree nor agree
- あまりあてはまらない Disagree
- まったくあてはまらない Strongly disagree

14. 6. 嫌な出来事があったとき、今の経験から得られるものを探す。 When I am faced with unpleasant situations, I try to gain something from those experiences. *

Mark only one oval.

- よくあてはまる Strongly agree
- ややあてはまる Agree
- どちらともいえない Neither disagree nor agree
- あまりあてはまらない Disagree
- まったくあてはまらない Strongly disagree

15. 7. 自分の考えや気持ちがよくわからないことが多い。 I often fail to understand my own feelings or thoughts. *

Mark only one oval.

- よくあてはまる Strongly agree
- ややあてはまる Agree
- どちらともいえない Neither disagree nor agree
- あまりあてはまらない Disagree
- まったくあてはまらない Strongly disagree

16. 8. 自分は体力がある方だ。 I have enough stamina. *

Mark only one oval.

- よくあてはまる Strongly agree
- ややあてはまる Agree
- どちらともいえない Neither disagree nor agree
- あまりあてはまらない Disagree
- まったくあてはまらない Strongly disagree

17. 9. 努力することを大事にする方だ。 I value working hard. *

Mark only one oval.

- よくあてはまる Strongly agree
- ややあてはまる Agree
- どちらともいえない Neither disagree nor agree
- あまりあてはまらない Disagree
- まったくあてはまらない Strongly disagree

18. 10. 人の気持ちや、微妙な表情の変化を読み取るのが上手だ。 I am good at detecting others' feelings or changes in facial expressions. *

Mark only one oval.

- よくあてはまる Strongly agree
- ややあてはまる Agree
- どちらともいえない Neither disagree nor agree
- あまりあてはまらない Disagree
- まったくあてはまらない Strongly disagree

19. 11. つらいことでも我慢できる方だ。 I can handle difficult experiences well. *

Mark only one oval.

- よくあてはまる Strongly agree
- ややあてはまる Agree
- どちらともいえない Neither disagree nor agree
- あまりあてはまらない Disagree
- まったくあてはまらない Strongly disagree

20. 12. 決めたことを最後までやりとおすことができる。　I can carry out decisions through to the end. *

Mark only one oval.

- よくあてはまる Strongly agree
- ややあてはまる Agree
- どちらともいえない Neither disagree nor agree
- あまりあてはまらない Disagree
- まったくあてはまらない Strongly disagree

21. 13. 思いやりを持って人と接している。　I treat someone with consideration. *

Mark only one oval.

- よくあてはまる Strongly agree
- ややあてはまる Agree
- どちらともいえない Neither disagree nor agree
- あまりあてはまらない Disagree
- まったくあてはまらない Strongly disagree

22. 14. 困難な出来事が起きても、どうにか切り抜けることができると思う。　I think that I can wriggle out when faced with a difficult problem. *

Mark only one oval.

- よくあてはまる Strongly agree
- ややあてはまる Agree
- どちらともいえない Neither disagree nor agree
- あまりあてはまらない Disagree
- まったくあてはまらない Strongly disagree

23. 15. 交友関係が広く、社交的である。I am an outgoing person and have a wide circle of friends. *

Mark only one oval.

- よくあてはまる Strongly agree
- ややあてはまる Agree
- どちらともいえない Neither disagree nor agree
- あまりあてはまらない Disagree
- まったくあてはまらない Strongly disagree

24. 16. 人と誤解が生じたときには積極的に話をしようとする。 When misunderstandings arise with others, I willingly have more talks. *

Mark only one oval.

- よくあてはまる Strongly agree
- ややあてはまる Agree
- どちらともいえない Neither disagree nor agree
- あまりあてはまらない Disagree
- まったくあてはまらない Strongly disagree

25. 17. 嫌な出来事が、どんな風に自分の気持ちに影響するか理解している。 I understand how unpleasant things influence my feelings. *

Mark only one oval.

- よくあてはまる Strongly agree
- ややあてはまる Agree
- どちらともいえない Neither disagree nor agree
- あまりあてはまらない Disagree
- まったくあてはまらない Strongly disagree

26. 18. 嫌な出来事があったとき、その問題を解決するために情報を集める。When I am faced with unpleasant situations, I try to gather information to solve the problem. *

Mark only one oval.

- よくあてはまる Strongly agree
- ややあてはまる Agree
- どちらともいえない Neither disagree nor agree
- あまりあてはまらない Disagree
- まったくあてはまらない Strongly disagree

27. 19. 嫌なことがあっても、自分の感情をコントロールできる。 I can control my feelings even if there is a disagreement. *

Mark only one oval.

- よくあてはまる Strongly agree
- ややあてはまる Agree
- どちらともいえない Neither disagree nor agree
- あまりあてはまらない Disagree
- まったくあてはまらない Strongly disagree

28. 20. 自分は粘り強い人間だと思う。I think I have perseverance. *

Mark only one oval.

- よくあてはまる Strongly agree
- ややあてはまる Agree
- どちらともいえない Neither disagree nor agree
- あまりあてはまらない Disagree
- まったくあてはまらない Strongly disagree

29. 21. 他人の考え方を理解するのが比較的得意だ。 I am good at understanding others' ways of thinking. *

Mark only one oval.

- よくあてはまる Strongly agree
- ややあてはまる Agree
- どちらともいえない Neither disagree nor agree
- あまりあてはまらない Disagree
- まったくあてはまらない Strongly disagree

コロナウイルス感染症流行下での健康行動Health behavior under COVID-19 Pandemic

コロナウイルス感染症流行下での対処行動と精神的健康面への影響に関する次の21項目について、それぞれあなたに最も当てはまると思う回答を１つ選んで、それぞれの行動を行う頻度を示してください。
The following 21 questions contain your preventive measures of the COVID-19 pandemic and its effects on your mental health status. Please answer to what extent are you engaging in /experiencing the following items.

30. 1. 公共の場でマスクを着用する。 Wear a mask in public. *

Mark only one oval.

- いつもある Always
- 時々ある Often
- あまりない Sometimes
- 全くない Never

31. 2. 自分の呼吸器症状（咳、鼻水、発熱、喉の痛みまたは呼吸困難）に注意する。 Self-monitor for respiratory symptoms (e.g., cough, runny nose, fever, sore throat, dyspnea). *

Mark only one oval.

- いつもある Always
- 時々ある Often
- あまりない Sometimes
- 全くない Never

32. 3. 呼吸器症状に対するエチケットを守る（例：咳やくしゃみをマスクやハンカチで覆ったり手洗いを頻回に行うなど）。 Follow respiratory hygiene recommendations (e.g., covering coughs or sneezes by wearing face masks or handkerchief, washing hands often). *

Mark only one oval.

- いつもある Always
- 時々ある Often
- あまりない Sometimes
- 全くない Never

33. 4. 手で顔（特に目、口、鼻）を触らないようにする。 Avoid hand to face (especially, eyes, mouth, and nose) contact. *

Mark only one oval.

- いつもある Always
- 時々ある Often
- あまりない Sometimes
- 全くない Never

34. 5. 定期的に体温を測定する。 Check body temperature regularly. *

Mark only one oval.

- いつもある Always
- 時々ある Often
- あまりない Sometimes
- 全くない Never

35. 6. 公共の場で物に触れた後は手を洗う、または消毒剤を使う。Wash hands or use hand sanitizer after touching objects and surfaces in public. *

Mark only one oval.

- いつもある Always
- 時々ある Often
- あまりない Sometimes
- 全くない Never

36. 7. 共有物品の表面を拭いたり消毒したりする。Clean and disinfect shared objects and surfaces. *

Mark only one oval.

- いつもある Always
- 時々ある Often
- あまりない Sometimes
- 全くない Never

37. 8. 帰宅後すぐにうがいする。Gargle immediately after returning home. *

Mark only one oval.

- いつもある Always
- 時々ある Often
- あまりない Sometimes
- 全くない Never

38. 9. 海外または国内の旅行を必要最低限にする。 Limit international or domestic travel only to the essential. *

Mark only one oval.

- いつもある Always
- 時々ある Often
- あまりない Sometimes
- 全くない Never

39. 10. 公共交通機関の使用を必要最低限にする。 Limit usage of public transportation only to the essential. *

Mark only one oval.

- いつもある Always
- 時々ある Often
- あまりない Sometimes
- 全くない Never

40. 11. 3つの密（密閉空間、密集場所、密接場面）を避ける。 Avoid crowded, closed, and close-contact settings. *

Mark only one oval.

- いつもある Always
- 時々ある Often
- あまりない Sometimes
- 全くない Never

41. 12. 人との会合を必要最小限にする。 Limit gatherings only to the essential. *

Mark only one oval.

- いつもある Always
- 時々ある Often
- あまりない Sometimes
- 全くない Never

42. 13. 公共エリアでの社会的距離（2メートル）を維持する。 Maintain social distance (i.e., two meters) in public areas. *

Mark only one oval.

- いつもある Always
- 時々ある Often
- あまりない Sometimes
- 全くない Never

43. 14. 重症化リスクの高い人（例：高齢者）との接触を避ける。 Avoid contact with individuals at high risk for severe illnesses (e.g. elders). *

Mark only one oval.

- いつもある Always
- 時々ある Often
- あまりない Sometimes
- 全くない Never

44. 15. 政府や都道府県からの情報や勧告について最新の情報を得る。 Keep up with the latest information and recommendations from health authorities. *

Mark only one oval.

- いつもある Always
- 時々ある Often
- あまりない Sometimes
- 全くない Never

45. 16. 政府の身体的/社会的距離（ステイホーム）の指令/要求に従う。 Follow the government's physical/social-distancing (stay-home) orders/requests. *

Mark only one oval.

- いつもある Always
- 時々ある Often
- あまりない Sometimes
- 全くない Never

46. 17. 社会的距離（ステイホーム）指令に従うことにより、ストレスやうつ症状を感じる。 Have stress and/or symptoms of depression due to following the social distancing (stay home) orders. *

Mark only one oval.

- いつもある Always
- 時々ある Often
- あまりない Sometimes
- 全くない Never

47. 18. 社会から隔離されていると感じる。 Feel alienated from society. *

Mark only one oval.

- いつもある Always
- 時々ある Often
- あまりない Sometimes
- 全くない Never

48. 19. ストレスを軽減するために工夫している。 Use specific measures to reduce stress. *

Mark only one oval.

- いつもある Always
- 時々ある Often
- あまりない Sometimes
- 全くない Never

49. 20. 身体活動を維持している（運動するなど）。 Maintain physical activities (have some exercise). *

Mark only one oval.

- いつもある Always
- 時々ある Often
- あまりない Sometimes
- 全くない Never

50. 21. 他の人にサポートを求める（家族/友人/学校/公共機関など）。 Seek support from others (e.g. family/friends/school/public authorities). *

Mark only one oval.

- いつもある Always
- 時々ある Often
- あまりない Sometimes
- 全くない Never

コロナウイルス感染症流行が与える影響（6項目）について、あなたに最も当てはまると思う回答を１つ選んで下さい。
The following 6 questions contain the impact of COVID-19 pandemic on different aspects. Please answer to what extent it had impacted you on each aspect.

51. 1. コロナウイルス感染症の流行は、あなたの日常生活や仕事(アルバイトも含む)勉強などにどの程度影響を与えましたか？ To what degree the COVID-19 pandemic affected your daily life and work (including part-time job) /study. *

Mark only one oval.

- とてもある Strongly
- 中程度ある Moderately
- わずかにある Slightly
- まったくない Not at all

52. 2. コロナウイルス感染症の流行はあなたの経済状況にどの程度影響しましたか？ To what degree the COVID-19 pandemic affected you financially. *

Mark only one oval.

- とてもある Strongly
- 中程度ある Moderately
- わずかにある Slightly
- まったくない Not at all

53. 3. コロナウイルス感染症の流行による学業や研究の遅れについて、あなたはどの程度心配していますか？ To what degree are you worried about academic (study/research) delays due to the COVID- 19 pandemic. *

Mark only one oval.

- とてもある Strongly
- 中程度ある Moderately
- わずかにある Slightly
- まったくない Not at all

54. 4. コロナウイルス感染症の流行があなたの人生に与える影響を克服する自信はどの程度ありますか？ To what degree do you have the confidence to overcome the impact of the COVID-19 on your life. *

Mark only one oval.

- とてもある Strongly
- 中程度ある Moderately
- わずかにある Slightly
- まったくない Not at all

55. 5. あなたの住んでいる地域ではCOVID-19感染予防管理が十分行われていると思いますか？ Do you think COVID-19 infection prevention management is adequate in your area? *

Mark only one oval.

- 非常にそう思う Strongly agree
- そう思う Agree
- どちらとも言えない Uncertain
- そう思わない Disagree
- 全くそうは思わない Strongly disagree

56. 6. あなたの大学ではCOVID-19感染予防管理が十分行われていると思いますか？ Do you think your university has adequate COVID-19 infection prevention management? *

Mark only one oval.

- 非常にそう思う Strongly agree
- そう思う Agree
- どちらとも言えない Uncertain
- そう思わない Disagree
- 全くそうは思わない Strongly disagree

コロナウイルス流行下で困っていることFacing problems under the coronavirus pandemic

57. その他、コロナウイルス流行下で特に困っていることがあればお書きください。In addition, please write if you are facing any problems during the coronavirus pandemic.

コントロール感と自己効力感の認知尺度 Perceived control and self-efficacy

次のコントロール感に関する11の質問についてそれぞれあなたに最も当てはまると思う回答を１つ選んで下さい。（＊留学生の方は自国での状況についてお答えください）
Please answer the following 11 questions about perceived control by clicking the statement which you feel most applicable. (* If you are an international student, please answer according to the situation in your own country)

58. 1. あなたは近所に影響を与える決定に影響を及ぼすことができると感じますか？ Do you feel you can influence decisions that affect your neighborhood? *

Mark only one oval.

- 非常にそう思う Strongly agree
- そう思う Agree
- どちらとも言えない Uncertain
- そう思わない Disagree
- 全くそうは思わない Strongly disagree

59. 2. 協働することによって、私の近所の人々は、近所に影響を与える決定に影響を及ぼすことができる。 By working together, people in my neighborhood can influence decisions that affect the neighborhood? *

Mark only one oval.

- 非常にそう思う Strongly agree
- そう思う Agree
- どちらとも言えない Uncertain
- そう思わない Disagree
- 全くそうは思わない Strongly disagree

60. 3. あなたはあなたの地域に影響を及ぼす決定に影響を与えることができると感じますか？ Do you feel you can influence decisions that affect your local area? *

Mark only one oval.

- 非常にそう思う Strongly agree
- そう思う Agree
- どちらとも言えない Uncertain
- そう思わない Disagree
- 全くそうは思わない Strongly disagree

61. 4. 協働することによって、私の地域の人々は、地域に影響を与える決定に影響を及ぼすことができる。 By working together, people in my area can influence decisions that affect the local area? *

Mark only one oval.

- 非常にそう思う Strongly agree
- そう思う Agree
- どちらとも言えない Uncertain
- そう思わない Disagree
- 全くそうは思わない Strongly disagree

62. 5. 私のような人々は、政府が行うことに何も言うことはない。 People like me have no say in what the government does. *

Mark only one oval.

- 非常にそう思う Strongly agree
- そう思う Agree
- どちらとも言えない Uncertain
- そう思わない Disagree
- 全くそうは思わない Strongly disagree

63. 6. 政府は一般的に私のような人々を公正に扱う。 The government generally treats people like me fairly. *

Mark only one oval.

- 非常にそう思う Strongly agree
- そう思う Agree
- どちらとも言えない Uncertain
- そう思わない Disagree
- 全くそうは思わない Strongly disagree

64. 7. 私の投票は選挙結果に何の影響もない。 My vote makes no difference to the outcome of an election. *

Mark only one oval.

- 非常にそう思う Strongly agree
- そう思う Agree
- どちらとも言えない Uncertain
- そう思わない Disagree
- 全くそうは思わない Strongly disagree

65. 8. どの政党が政権を持つかは私たちの生活に影響を及ぼすのでとても重要である。 It really matters which party is in power, because it will affect our lives. *

Mark only one oval.

- 非常にそう思う Strongly agree
- そう思う Agree
- どちらとも言えない Uncertain
- そう思わない Disagree
- 全くそうは思わない Strongly disagree

66. 9. 近い将来私にとって物事が良くなると予期している。 I expect things to get better for me in the foreseeable future. *

Mark only one oval.

- 非常にそう思う Strongly agree
- そう思う Agree
- どちらとも言えない Uncertain
- そう思わない Disagree
- 全くそうは思わない Strongly disagree

67. 10. 私の生活/人生に影響する決定について自分がコントロールできる分量に満足している。 I am satisfied with the amount of control I have over decisions that affect my life. *

Mark only one oval.

- 非常にそう思う Strongly agree
- そう思う Agree
- どちらとも言えない Uncertain
- そう思わない Disagree
- 全くそうは思わない Strongly disagree

68. 11. あなたは新型コロナウイルスに感染しないようにどの程度管理していると思いますか？ How much do you control to avoid infection from the virus? *

Mark only one oval.

- コントロールしてない No control
- 少しコントロールしてる A little control
- かなりコントロールしてる A great deal of control

ライフスタイル Lifestyle

あなたのライフスタイルについて3 項目の質問に回答をクリックしてください。 Please click your answer(s) in 3 questions on your lifestyle.

69. 1. 通常1日に何時間睡眠をとりますか? How many hours do you usually sleep in a day? *

70. 2. お酒を飲みますか？ Do you drink alcohol? *

Mark only one oval.

- いいえ Never
- 時々飲む Sometimes
- 毎日飲む Every day

71. 3. 喫煙しますか？ Do you smoke? *

Mark only one oval.

- いいえ Never
- はい Yes

基礎情報 Basic Demographic Questionnaire

あなたの基礎情報について16項目の質問に回答をクリックしてください。
Please click your answer(s) in 16 questions on your basic information.

72. 1. あなたの学年を教えてください。 The school year of enrolled program *

Mark only one oval.

- 1年生 The first year
- 2年生 The second year
- 3年生 The third year
- 4年生 Fourth year
- 5年生 Fifth year
- Other: ____________

73. 2. あなたの満年齢（歳）を教えてください。 Age

____

74. 3. 専攻 Major *

Mark only one oval.

- 看護専攻 Nursing
- 医学専攻 Medical
- 歯学専攻 Dental
- Other: ____________

75. 4. 学部/大学院 Enrolment program *

Mark only one oval.

- 学部生 Undergraduate program
- 大学院生 Graduate program

76. 5. 性別 Sex

Mark only one oval.

- 男性 Male
- 女性 Female
- Other: ____________

77. 6. あなたは留学生ですか？ Are you international students? *

Mark only one oval.

- はい Yes
- いいえ No

78. 7. 国籍 Nationality

Mark only one oval.

- 日本 Japan
- アメリカ United states
- 中国 China
- インドネシア Indonesia
- ミャンマー Myanmar
- タイ Thailand
- ベトナム Vietnam
- ガーナ Ghana
- オーストラリア Australia
- 香港 Hongkong
- Other: ______________________

79. 8. 人種 （複数回答可）Race (multiple choice is acceptable)

Check all that apply.

- アジア系 Asian, Native Hawaiian or Other Pacific Islander
- 白人 Caucasian
- アフリカ系 African, Black or African American
- ヒスパニック Hispanic/ Latinx
- アイヌ系/American Indian or Alaskan Native
- 回答したくない prefer not to answer

Other: ______________________

80. 9. 宗教 Religious

Mark only one oval.

- 仏教 Buddhism
- 神道 Shintoism
- キリスト教 Christianity
- イスラム教 Islam
- ヒンズー教 Hinduism
- なし None
- Other: ____________________

81. 10. 現在仕事（アルバイト）をしていますか？ Are you currently working (part-time job)?

Mark only one oval.

- 全くしていない No
- 週5時間未満 Less than 5 hours a week
- 週5～10時間未満 From 5 to less than 10 hours a week
- 週10~20時間未満 From 10 to less than 20 hours a week
- 週20時間~40時間未満 From 20 to less than 40 hours a week
- 週40時間以上 40 hours or more a week
- Other: ____________________

82. 11. あなたは今までにコロナウイルス陽性と診断されたことがありますか？ Have you ever been diagnosed with coronavirus positive?

Mark only one oval.

- はい Yes
- いいえ No

83. 12. あなたの家族/同僚/近い友人が今までにコロナウイルス陽性と診断されたことがありますか？ Have your family / colleagues / close friends ever been diagnosed with coronavirus positive?

Mark only one oval.

- はい Yes
- いいえ No

84. 13. 実習も含めコロナ患者に対応したことがありますか？ Have you taken care of patients with COVID-19, including practical training?

Mark only one oval.

- はい Yes
- いいえ No

85. 14. 現在コロナ患者の直接ケアを継続的に行っていますか？ Are you currently working with patients with COVID-19?

Mark only one oval.

- はい Yes
- いいえ No

86. 15. 同居 Are you currently living with somebody (housemate)?

Mark only one oval.

- 一人暮らし Alone
- 他の人と同居してる Live with somebody

87. 16. 定期的に受診が必要な慢性疾患はありますか？ Do you have a chronic condition that requires regular medical checkups? *

Mark only one oval.

- はい Yes
- いいえ No

以上で調査は終了です。ご協力ありがとうございました。 End of Survey. Thank you for your participation.

This content is neither created nor endorsed by Google.

Google Forms
